# Emergent factors in Eating Disorders in childhood and preadolescence

**DOI:** 10.1186/1824-7288-36-49

**Published:** 2010-07-08

**Authors:** Leonardo Sacrato, Alessandro Pellicciari, Emilio Franzoni

**Affiliations:** 1 Child Neuropsychiatry Unit, Sant'Orsola Malpighi Hospital, University of Bologna, Italy

## Abstract

We have reviewed the literature related to the current advances in comprehension of Eating Disorders (ED) in childhood and preadolescence. The state of art regarding the psychodynamic models concerning the onset of ED are explained. DSM-IV and ICD-10 criteria are discussed, pointing out their little value in the characterization of early eating difficulties. Historic and new diagnostic classifications are displayed in detail. We provided a clearer description of subclinical patterns. Finally we focus on the key role of the paediatrician in detecting and managing parental concerns regarding feeding.

## Introduction

Parents are often concerned about eating disturbances in young children and frequently turn to their family physician for advice. It has been estimated that 25% of normally developing children can show difficulties with food [1,2] and that this percentage increases to 35% when the population is restricted to children with development-related problems (prematurity, specific deficits) [3]. Pediatricians report a prevalence of 5-10% of severe eating disturbances like persistent food refusal; these conditions can result in a failure to thrive and require hospitalization [2].

In particular, failure to thrive is determined by non-organic causes in 50-58% of cases [4].

On the other hand, 6% of infants in the 6-15 months range of age are reported by their caregivers to have feeding difficulties, arising to about 25-40% during latter phases of development [3,5]. This percentages include aspecific patterns (colic, vomiting, slow-feeding) as well more structured restrictive or hyperphagic behaviors, and even well-codified disorders such as pica and mericism.

Marchi and Cohen traced maladptative eating patterns of 800 toddlers during a 10-years follow-up. Picky eating and selective alimentation were found to be predictors of a subsequent Anorexia Nervosa (AN), while prospective risks for a later Bulimia Nervosa (BN) implicated pica and irregular meals [6].

Moreover, the historic studies of Anna Freud and the investigations of C. Keller detected a connection between feeding and eating disturbances of infancy and latter onset of behavioral and personality disorders [7,8].

Thus, a special awareness about feeding-related problems is needed by the physician who firstly deals with the child: the pediatrician.

## Clinical aspects and psychodynamic models

In the last decades, investigations upon early Eating Disorders (ED) have been encouraged by recent progresses of Infant Research. This methodologies of observation study the child's behavior in his own environment in a detailed and microanalytic way. It is now known that infants are able to call the mother's attention with the cry, gazes and gestures in order to stimulate her appropriate reactions.

This emotive resonance allows the infant to start organizing his bodily experiences and a child who has a supportive relationship with his parents is likely to develop a working model of self as worthy of love [9].

The psychical development is thus permitted by the caretaker's ability of executing an affective link with the son's mood states. Satiety and hunger are associated with positive or negative feelings that amplify their connotations. The reiterating cycle of physiological needs (hunger), followed by their satisfaction (being fed by the mother) determine in the child a familiar regulation of states; failure in attachment process or a lack of correspondence between infant's needs and parental answers can compromise the successful regulation of child's hunger. Cooper [10] argued that a disturbance in the caregiver-infant relationship, where the caregiver responses are not appropriate with the infant's internal state, may result in deficit of self-awareness, self-concept and inability to distinguish hunger and satiety from other needs and discomforts.

Therefore nutrition can be a highly pleasant and reassuring experience, or it can be perceived as a struggle for autonomy and a way to affirm the Self individualization.

Mothers who feed adequately their sons experience pride and satisfaction. On the other hand, as their food becomes refused by the infant, they feel inadequate and frustrated. Therefore toddlers learn to use nutrition as a way of communicating their own personality while feeding problems can evoke caregiver distress, worries and anxiety [11].

Refusals of food can have a sudden or a progressive onset. Due to the panic arisen, caregivers (and particularly mothers) can use coercive and forceful mealtime strategies. Feeding acquires a negative atmosphere that overloads the two contenders with an important distress.

The mutual interactions between parents and son are a regulative system where each of the two components affects the other's behavior, by permitting or denying the psychological growth, protecting from risk factors or possibly transmitting negative influences [12,13]. By referring to parents, we wish to underline the important figure of the father, a role that has been ignored by the international literature for decades. By his presence, the father acts as the external reality who breaks the strict link between mother and child, allowing the child individualization, his awareness about the limits of his own body and his conscience that another world, different by the maternal embrace, exists.

Recent studies [14] pointed out that the "Male's function" chiefly consists of helping his mate in keeping the levels of sufferance tolerable. With regard to attachment, the father both acts as a compensative factor (operating when the mother's caregiving style is difficult or poor) and as a mutative factor of the internal models that characterize his wife's attachment style, improving and possibly stopping pathological dyadic relationships. Therefore the paternal figure can play an important modulating role both in the marital relationship and in the mother-son interactions.

## Diagnostic classifications

In order to define eating psychopathologies the diagnostic classifications have to consider each of the following: child, attachment figure, relational dynamics, developmental phase. The first attempt was performed by Anna Freud [15]; she considered organic ED, difficulties due to differences between parental feeding program and child's needs, and neurotic eating disorders (determined by hostile feelings in the relationship with the caregiver, transferred on the food that he symbolizes). Afterwards, Kreisler [16] created a diagnostic guideline that considered the different forms of infantile anorexia as failures of the interactive maternal functioning that modulates the child's psychosomatic poise. He distinguished: simple anorexia of infancy (characterized by oppositive behaviors following trigger events such weaning) and complex anorexias of the 1^st ^year of life (phobic or depressive forms with psychosomatic disorganization). In his diagnostic scheme he considered several pathogenic influences: chronic affective lack, that can lead to high risk eating patterns like severe forms of anorexia, psychogenic vomit, rumination; duress, intrusiveness and maternal rigidity that can cause eating active refusal; separations, discontinuity, inconsistency in life and educational styles in the caregiving environment, usually occurring in high psychosocial risk families. Instead, Woolston's classification [17] is focused on the peculiar characteristics of the child, mother and their interactions. It included three types of non-organic failure to thrive: type 1, that is a reactive disturbance of attachment (onset: 8 months), characterized by a depressed, socially isolated mother and by an apathetic and socially retired child (this aspect is reflected during meals with a lack of reciprocity); type 2, a malnutrition with an inadequate diet due to cultural differences or economic problems; type 3 or pathologic refusal of food, having onset between 6-16 months, determined by the infant's drive for autonomy and characterized by hostile relationships with the caregiver.

In 1994, DSM-IV TR [4] for the first time reserved a specific chapter distinguishing ED occurring during infancy from those arising in adolescence. Although scarce, this section included: pica, rumination disorder and feeding disorder of infancy or early childhood. This latter condition considers every eating pattern characterized by eating difficulties with loss of weight or inability in weight gaining. Also the ICD10 appears lacking in specificity, as it includes only pica and the generic feeding or ED of infancy and early childhood. Since neither of these latter classifications consider specific aspects as the quality of attachment relationships and their impact on nutrition, the subject's emotional state or the family environment, further nosographic systems have been proposed [18]. These new clinical-developmental classifications are intended as a means of integrating or replacing DSM-IV and ICD-10 by focusing on particular diagnostic subgroups emerging from the clinical observation of the relational dynamics associated with early-onset ED. Among these diagnostic guidelines, the one proposed by I. Chatoor [19] appears useful in the first phases of development, as it distinguishes:

- Feeding disorder of homeostasis (0-3 months): early difficulties in reaching and maintaining the state of calm alertness necessary for healthy feeding; the child appears irritable. It can preclude to further troubles during the weaning.

- Feeding disorder of attachment (2-8 months), which is characterized by a lack of social reciprocity (visual engagement, smiling, babbling during feeding) and a failure to grow.

- Separation feeding disorder or infantile anorexia (6 months - 3 years): the child does not communicate hunger and has no interest in food. The disorder has to be intended as a struggle for autonomy; the mother tends to react with rigid and intrusive behaviors.

- Sensory food aversions: a refusal to eat specific foods with certain tastes, smells, textures or appearances during the introduction of a new food.

- ED associated with a medical condition: there is a failure to gain weight or

weight is lost. Treatment of the medical condition may improve but not eliminate the problem.

- Post-traumatic feeding disorder: the child may refuse food following a traumatic or related event such as the insertion of a nasogastric tube, an episode of choking, severe vomiting or aspiration. He or she may show intense resistance if reminded of the traumatic event.

A further and recent classification has been proposed by the Workgroup for Classification for Eating Disorders in Children and Adolescent (WCEDCA) [20], and presents a detailed description of eating disorders focusing on the prepuberal age.

-Food avoidance emotional disorder (FAED), characterized by emotional-based refusal of food without the weight and shape concerns typical of Anorexia Nervosa,

-Functional dysphagia, that describes swallowing difficulties associated with a fear of choking leading to reduction of food intake,

-Pervasive refusal syndrome, that is a profound and pervasive refusal to eat, walk, talk or engage in self care,

-Selective eating, whose pattern is constituted by restricted choices of food and refusal of new foods,

-Anorexia Nervosa,

-Bulimia Nervosa.

In order to plan adequate interventions, the ability of the pediatrician to suspect and distinguish even these subclinical patterns is very important.

Literature reports data about both the increasing prevalence of FAED [21] and the worse long-term outcome of patients affected by FAED when compared to children who experience emotional malaises without involvement of the eating-related area [22]. Finally, there is some evidence that these conditions may represent precursors of a further full-blown Anorexia [23] and the infantile equivalent of a Somatoform Disorder [24].

## Conclusions

Clinical experience and recent literature acquisitions determined a re-conceptualization of ED, that have to be understood and managed with an integrated perspective: clinical/biological and psychological aspects, social environment, cultural influences and relationships with the Other (both belonging to the family nucleus or not) should all be considered. A child's malaise that is intolerable, or cannot be imagined and contained by parents, is expressed trough the body and revealed by symptoms. Thus the body acts a communicative role and is lived as a relationship with the Other. Symptoms can reveal the presence of more complex problems whose expression may range from slight changes in feeding functions to a pervasive refusal of food.

The pediatrician plays a crucial role since he can: contain the parental anxiety induced by the child's feeding difficulties; promote the inclusion of the paternal figure in the mother and child dyadic system; encourage the mother to respect the son's attitudes towards nutrition; help the mother to tolerate the frustration that the phase of weaning can determine; deter dysfunctional behaviors (like distracting the infant and then forcing him to eat as he lowers the attention); explicating the meaning of each developmental acquisition.

If the physician notices high risk behaviors that can eventually belong to one of the described psychopathological patterns, we recommend the intervention of a specialized Center.

The therapeutic approach will be focused firstly on the detection of the malaise that underlies the symptoms. Subsequently, a patient-focused therapeutic plan will be proposed in order to obtain the management and the resolution of the background dynamics that determine symptoms onset.

Periodic follow-up observations have to be organized in order to assess the young patient and his family even after the resolution of the acute phase.

## Competing interests

The authors declare that they have no competing interests.

## Authors' contributions

LS, AP and EF contributed equally to this work. All authors have read and approved the final manuscript.
